# Importance of diffuse scattering for materials science

**DOI:** 10.1107/S2052252516013889

**Published:** 2016-08-31

**Authors:** Hiroshi Sawa

**Affiliations:** a Department of Applied Physics, School of Engineering, Nagoya University, Nagoya, Aichi, Japan

**Keywords:** high-*T*_c_ superconductors, HgBa_2_CuO_4+δ_, diffuse X-ray scattering, materials science

## Abstract

Recent studies highlighting the importance of diffuse scattering for materials science are presented.

A number of recent studies have highlighted the importance of diffuse scattering for materials science. These include a paper by Welberry & Goossens (2016 ▸) in this issue of **IUCrJ** on the high-*T*_c_ superconductor HgBa_2_CuO_4+δ_ and several studies of the exotic properties of 6*H*-Ba_3_CuSb_2_O_9_.

In order to understand the electronic state of a cuprate high-*T*_c_ superconductor, information on the detailed structural parameters is required. Because the HgBa_2_CuO_4+δ_ system has a high transition temperature, it is an important system for studying the electronic state using structural information. However, this system exhibits a diffuse scattering pattern as a result of its intrinsic structural imperfection. The existence of incompleteness in a crystal (*e.g.* a lattice defect) is frequently ignored and physical properties are instead correlated with an average periodic structure. However, when imperfections in the local structure have a large influence on physical properties, it is necessary to understand symmetry and periodicity breaking. From this point of view, an analysis of diffuse scattering is important for structural physics.

The paper by Welberry & Goossens (2016 ▸) on HgBa_2_CuO_4+δ_ is a very interesting re-examination of results previously published by Izquierdo *et al.* (2011 ▸). The ‘apparent valence’ or ‘bond-valance sum’ method used by Welberry & Goossens has proven to be a valuable and interesting tool for guiding the development of disorder models and verifying the results. The work is an excellent demonstration of how to analyze the diffuse scattering of experimental diffraction patterns. Computer simulation of model structures has become a powerful and well accepted technique for aiding the interpretation and analysis of diffuse scattering patterns. The final model includes the displacement shifts of Ba, Hg and O. In general, researchers believe that oxygen shifts have little direct effect on the diffraction pattern since the scattering from the oxygen is very weak in comparison to the shifts of heavy ions. However, this paper clearly shows that displacement shifts are the key to understanding the incomplete structure since they are the main driving force that enables the oxygen to achieve a normal valence. Even fine details of the diffraction pattern could be reproduced, allowing the establishment of a semi-quantitative model merely by visual comparison of experimental and calculated diffraction patterns.

David A. Keen and Andrew L. Goodwin have described how correlated disorder has clear crystallographic signatures that map to the type of disorder, irrespective of the underlying physical or chemical interactions and material involved. They noted that the study of diffuse scattering patterns for disordered states will help us to understand, control and exploit the disorder responsible for many interesting physical properties (Keen & Goodwin, 2015 ▸).

Recent studies (Katayama *et al.*, 2015 ▸) have clarified the possibility of an exotic quantum liquid state based on spin–orbital entanglement in 6*H*-Ba_3_CuSb_2_O_9_. The crystal structure of this system by averaged structural analysis was reported as centrosymmetric, consisting of a positive triangular lattice of Cu^2+^ (*S* = 1/2) at room temperature. In strongly correlated electron systems, orbital ordering usually appears at high temperatures in a process accompanied by a lattice deformation, called a static Jahn–Teller distortion. However, this system exhibits an absence of Jahn–Teller transition down to 20 mK and a dynamic spin state. To confirm this exotic ground state, experiments based on single-crystal samples are essential. However, this system is difficult to analyze by ordinary methods because of the existence of a characteristic honeycomb diffuse scattering pattern (see Fig. 1 ▸) (Nakatsuji *et al.*, 2012 ▸).

The single-crystal X-ray diffraction patterns of 6*H*-Ba_3_CuSb_2_O_9_ show strong, temperature-independent, diffuse scattering that is offset by (1/3, 1/3, 0) from the reciprocal lattice points. This indicates a three-sublattice, ferrielectric short-range order of Cu–Sb dumbbell orientations (up/down) in the *ab* plane. The broad diffuse scattering patterns indicate isotropic ∼10 Å domains consistent with the absence of second harmonic generation. We propose that this unusual chemical short-range order is formed as a result of frustrated electric dipole–dipole interactions between Cu–Sb dumbbells that emulate Ising spins on a triangular lattice. This short-range order is in turn frozen in place as the material is cooled during solid-state synthesis. By analysis of the characteristic diffuse scattering pattern in this system, we find that this frustration is imprinted in a nanostructured honeycomb lattice of Cu^2+^ ions that resists a coherent static Jahn–Teller distortion. The resulting two-dimensional random-bond spin-1/2 system on the honeycomb lattice has a broad spectrum of spin-dimer-like excitations and low-energy spin degrees of freedom that retain overall hexagonal symmetry.

The orbital energy, *i.e.* the interaction energy that lifts the orbital degeneracy and induces low-temperature orbital ordered states, is typically much higher than the spin energy owing to the cooperative Jahn–Teller effect, resulting in a weak correlation between spin and orbital. However, the orbital energy is reduced due to spatially separated CuO_6_ octahedra in the honeycomb lattice (Katayama *et al.*, 2015 ▸).

If the magnetic exchange interactions are enhanced to the point that they are comparable with the orbital energy, the interplay between spin and orbital would destabilize the conventional orbital-ordered state, leading to a novel spin–orbital entangled state. Indeed, such a spin–orbital correlation has been already pointed out using Huang scattering (Ishiguro *et al.*, 2013 ▸).

The analysis of diffuse scattering is thus very valuable for material scientists interested in local symmetry breaking. An analysis of local symmetry can highlight exotic physical properties clearly, but the electronic state has to be confirmed by multiangular experimental methods.

## Figures and Tables

**Figure 1:**
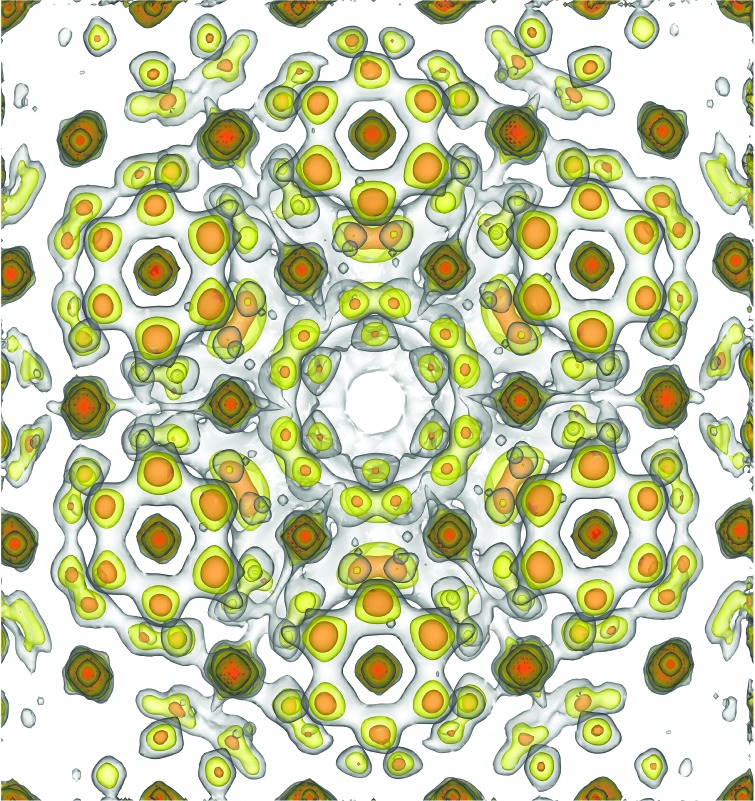
Honeycomb diffuse scattering pattern of 6*H*-Ba_3_CuSb_2_O_9_ (Nakatsuji *et al.*, 2012 ▸).

## References

[bb1] Ishiguro, Y., Kimura, K., Nakatsuji, S., Tsutsui, S., Baron, A. Q. R., Kimura, T. & Wakabayashi, Y. (2013). *Nat. Commun.* **4**, 2022. 10.1038/ncomms3022 23771213

[bb2] Izquierdo, M., Megtert, S., Colson, D., Honkimäki, V., Forget, A., Raffy, H. & Comès, R. (2011). *J. Phys. Chem. Solids*, **72**, 545–548.

[bb3] Katayama, N., Kimura, K., Han, Y., Nasu, J., Drichko, N., Nakanishi, Y., Halim, M., Ishiguro, Y., Satake, R., Nishibori, E., Yoshizawa, M., Nakano, T., Nozue, Y., Wakabayashi, Y., Ishihara, S., Hagiwara, M., Sawa, H. & Nakatsuji, S. (2015). *Proc. Natl Acad. Sci. USA*, **112**, 9305–9309. 10.1073/pnas.1508941112 PMC4522741 26170280

[bb4] Keen, D. A. & Goodwin, A. L. (2015). *Nature*, **521**, 303–309. 10.1038/nature14453 25993960

[bb5] Nakatsuji, S. K., Kuga, K., Kimura, R., Satake, N., Katayama, E., Nishibori, H., Sawa, R., Ishii, M., Hagiwara, F., Bridges, F., Ito, W., Higemoto, Y., Karaki, M., Halim, M., Nugroho, J. A., Rodriguez-Rivera, M. A., Green, C. & Broholm, (2012). *Science*, **336**, 559–563. 10.1126/science.1212154 22556246

[bb6] Welberry, T. R. & Goossens, D. J. (2016). *IUCrJ* **3**, 309–318. 10.1107/S2052252516010629 PMC5391853 28461892

